# Dielectric Surface-Based Biosensors for Enhanced Detection of Biomolecular Interactions: Advances and Applications

**DOI:** 10.3390/bios14110524

**Published:** 2024-10-30

**Authors:** Liangju Li, Jingbo Zhang, Yacong Li, Caixin Huang, Jiying Xu, Ying Zhao, Pengfei Zhang

**Affiliations:** 1 School of Pharmacy, Xinxiang Medical University, Xinxiang 453003, China; liliangju@iccas.ac.cn (L.L.); liyacong@iccas.ac.cn (Y.L.); huangcaixin@iccas.ac.cn (C.H.); 2 Beijing National Laboratory for Molecular Sciences, Key Laboratory of Analytical Chemistry for Living Biosystems, Institute of Chemistry, Chinese Academy of Sciences, Beijing 100190, China; jingbozh@iccas.ac.cn (J.Z.); xuiy@iccas.ac.cn (J.X.); 3 University of Chinese Academy of Sciences, Beijing 100049, China

**Keywords:** biomolecular interactions, dielectric surface, surface plasmon resonance, label-free, binding kinetics

## Abstract

Surface plasmon resonance (SPR) biosensors are extensively utilized for analyzing molecular interactions due to their high sensitivity and label-free detection capabilities. Recent innovations in surface-sensitive biosensors with dielectric surfaces address the inherent limitations associated with traditional gold surfaces, such as thermal effects and biocompatibility issues, which can impede broader applications. This review examines state-of-the-art biosensor configurations, including total internal reflection, optical waveguide, photonic crystal resonators, Bloch surface wave biosensors, and surface electrochemical biosensors, which can enhance analyte signals and augment the molecular detection efficiency at the sensor interface. These technological advancements not only improve the resolution of binding kinetics analysis and single-molecule detection but also extend the analytical capabilities of these systems. Additionally, this review explores prospective advancements in augmenting field enhancement and incorporating multimodal sensing functionalities, emphasizing the significant potential of these sophisticated biosensing technologies to profoundly enhance our understanding of molecular interactions.

## 1. Introduction

Elucidating molecular binding kinetics is pivotal for the screening of pharmacological agents, the detection of disease-specific biomarkers, and the delineation of biological processes at the molecular scale [1,2,3,4]. The kinetic parameters governing drug–target interactions, specifically the association and dissociation rates, are critical determinants of therapeutic efficacy and duration of action [4]. Contemporary research emphasizes the significance of drug–target residence time as a key factor influencing the sustained pharmacological activity of a drug, independent of its systemic concentration [5,6,7,8,9]. This paradigm shifts from evaluating the mere equilibrium binding affinity to considering the dynamic interplay of drug–target interactions under non-equilibrium conditions in vivo offers a more comprehensive understanding of drug behaviors [10,11]. Accordingly, incorporating drug–target residence time into the drug development process enhances the prediction of drug efficacy in physiological settings, facilitating the creation of more effective and precisely targeted therapeutic interventions [12,13,14].

**Figure 1:**
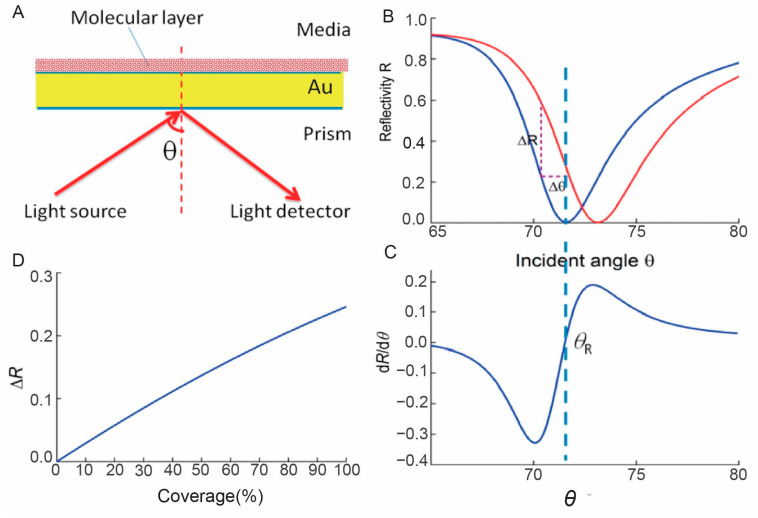
Stratified medium model for surface plasmon resonance (SPR). (**A**) Schematic of the stratified medium model; (**B**) SPR reflectivity curves without (blue) and with (red) a 10 nm polystyrene film; (**C**) slope of the reflectivity curve; (**D**) reflectivity changes with varying coverage of 5 nm protein molecules [15].

Surface plasmon resonance (SPR) relies on the excitation of surface plasmons at the interface between a metal and a dielectric. When light hits the metal at a specific angle, known as the resonance angle, surface plasmons are generated, leading to a sharp drop in reflectivity. Changes in the resonance angle or reflectivity can indicate molecular adsorption on the surface. This principle is described using the stratified medium model, which calculates reflectivity changes based on the thickness and refractive index of the adsorbed layers (Figure 1 gives an schematic illustration). SPR can sensitively detect variations in molecular coverage, as seen in reflectivity shifts due to different protein layer thicknesses [15]. SPR is a typical surface-sensitive biosensor and has become a powerful tool for quantifying molecular binding kinetics [16,17,18,19,20]. This technique capitalizes on surface plasmonic waves, with the field intensity concentrated within approximately 100 nm of the sensor surface [21,22,23,24,25,26]. This confinement effectively shields the measurements from interference caused by molecules and impurities in the bulk solution, making SPR exceptionally well suited for examining surface-bound interactions [21,22,23]. When used with surfaces modified with specific receptors, SPR facilitates the detailed study of ligand-binding dynamics in a label-free context. Furthermore, the significant enhancement (20–30 times) of the field near the sensor surface underpins the high sensitivity characteristic of SPR [27,28]. Advancing beyond the foundational capabilities of conventional SPR, SPR microscopy (SPRM) enhances the methodology by integrating spatially resolved imaging capabilities. SPRM employs high numerical aperture objectives to improve spatial resolution and reduce optical aberrations, issues often encountered with prism-based systems [28,29,30,31,32,33,34]. These advancements allow for the meticulous visualization and quantification of biomolecular interactions at the level of individual entities, thereby yielding deep insights into the spatial variability of molecular binding characteristics. Recently developed plasmonic scattering microscopy (PSM) has further advanced this field by pushing the SPR detection limit down to the single-protein level and facilitating high-throughput single-cell analysis. This newly developed technique employs out-of-plane scattering of surface plasmonic waves by analytes, which enhances label-free detection capabilities and spatial resolution. By delivering substantial enhancements in image clarity and resolution, PSM serves as a pivotal tool for detailed study in both single-molecule and single-cell domains [35,36,37].

Despite notable advancements, the application of SPR methodologies is somewhat restricted in certain pioneering areas due to limitations associated with the metallic (typically gold) surfaces employed. For instance, the requisite high-incident light intensities for label-free single-protein detection can cause significant heating of the gold surface, thereby constraining its use in prolonged single-protein analyses. Furthermore, gold surfaces are not inherently biocompatible and often necessitate specific modifications to facilitate cell attachment, complicating procedural workflows [38].

Dielectric surfaces have emerged as a promising alternative, offering several key advantages over traditional metallic SPR surfaces. First, dielectric materials, such as silicon dioxide (SiO₂) and titanium dioxide (TiO₂), exhibit lower thermal conductivity, which significantly reduces heat buildup during high-intensity light exposure, making them better suited for prolonged biomolecular studies. Additionally, dielectric surfaces are inherently more biocompatible, eliminating the need for complex surface modifications and enabling more straightforward integration into biological experiments. Recent advancements in electromagnetic wave-to-heat-to-electric conversion technologies, such as those demonstrated by Xiong et al. [39], have shown how structured materials and optimized design principles can significantly enhance energy localization and field intensity at the surface. These principles, when applied to dielectric surface-based biosensors, can lead to the ultra-sensitive detection of biomolecules, facilitating real-time, label-free molecular analysis. Dielectric-based sensors can also maintain strong field confinement near the surface, which is crucial for enhancing sensitivity. In fact, dielectric film coatings have demonstrated the potential to improve surface light field intensification beyond that of traditional SPR systems, paving the way for the ultra-sensitive detection of biomolecules with minimal signal degradation due to heat or surface incompatibility.

In this review, we start with a detailed exploration of the core principles and advanced instrumentation crucial for surface-sensitive techniques that use dielectric surfaces. We then discuss recent developments in this area. To conclude, we identify ongoing challenges and share our perspective on the prospects and potential applications of surface-sensitive biosensors.

## 2. Working Mechanisms of Dielectric Surface-Based Biosensors

### 2.1. Total Internal Reflection Biosensors

Total internal reflection (TIR) occurs when light traveling through a medium with a higher refractive index encounters an interface with a medium with a lower refractive index at an angle greater than the critical angle. Instead of passing through the interface, the light is completely reflected back into the higher refractive index medium. Despite this reflection, an evanescent field is generated at the interface in the lower refractive index medium, extending only a short distance (typically around 100–200 nm) from the surface. TIR is a longstanding optical technique that has been revitalized through integration with innovative detection principles. The cover glass is usually employed as the sensor chip to provide a dielectric sensor surface in TIR biosensors, and the evanescent waves excited by the TIR are employed as the surface light illumination to achieve surface-sensitive measurements similar to SPR [40,41,42].

**Figure 2:**
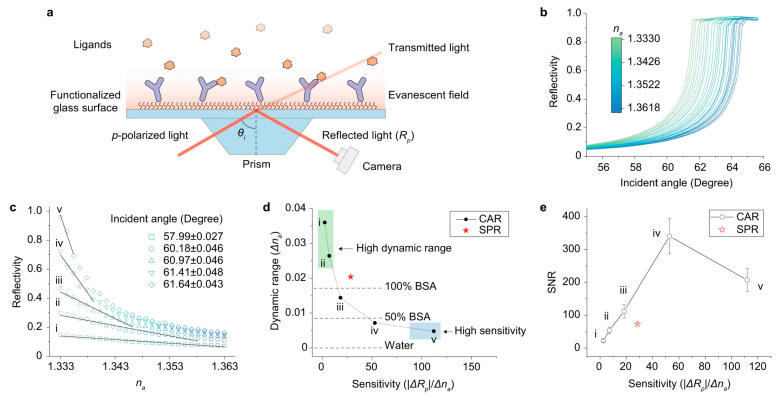
TIR-CAR biosensors. (**a**) Experimental setup and surface chemistry. (**b**) Measured reflectivity change as a function of incident angle. (**c**) Reflectivity as a function of sample refractive index at five representative incident angles (i–v). (**d**) TIR sensor sensitivity and dynamic range at the five representative angles in (**c**). (**e**) Signal-to-noise ratio comparison for SPR and TIR at the five representative angles in (**c**) [40].

TIR biosensors with a critical angle reflection configuration (TIR-CAR) have been used to monitor molecular binding behaviors on the surface and surface electrochemical processes [43]. As shown in Figure 2, the imaging system of TIR-CAR is implemented using a cover glass, which reflects incident light near the critical angle. The binding events on the glass surface alter the refractive index near the surface, leading to changes in the reflected light intensity, which is captured by a camera [40].

**Figure 3:**
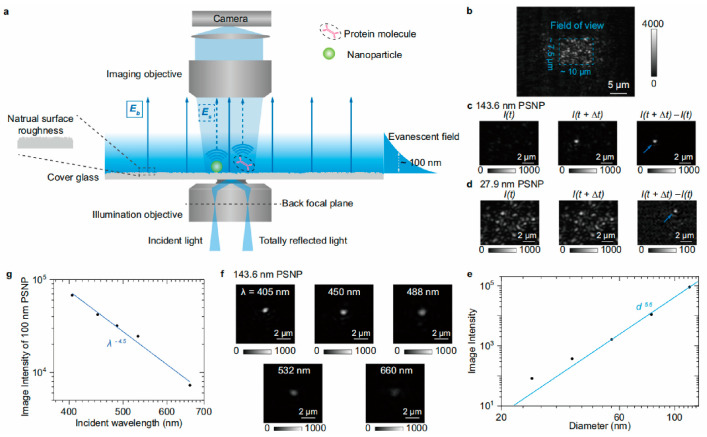
TIR-ESM biosensors for label-free single-protein imaging. (**a**) Optical setup schematic. (**b**) Raw image of a bare cover glass. (**c**,**d**) Images before and after binding of nanoparticles. (**e**) Image intensity versus particle diameter. (**f**) Images of nanoparticles with different incident wavelengths. (**g**) Image intensity versus incident wavelength [44].

With a scattering detection scheme, the TIR biosensors achieved the capability of label-free single-protein imaging [44,45]. As illustrated in Figure 3, the TIR with an evanescent scattering microscopic detection configuration (TIR-ESM) biosensor detects evanescent waves scattered by the individual proteins interfering with those scattered by the glass roughness. The evanescent waves scattered by the glass surface enhance the scattering signals of protein targets. In addition, the camera does not collect strong reflection light, thus allowing high-incident intensity to further enhance the signal-to-noise ratio [44]. Owing to these, this method allows for label-free single-protein imaging.

### 2.2. Surface Charge Distribution Biosensors

**Figure 4:**
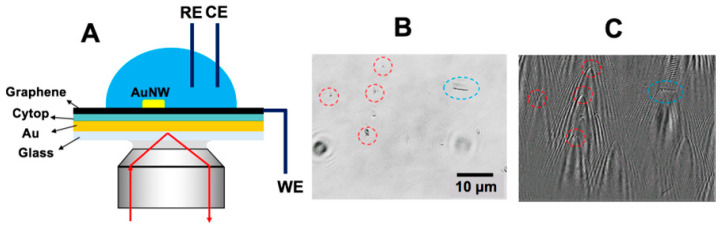
Surface charge distribution biosensors. (**A**) Experimental setup with a gold film coated with a Cytop dielectric layer and a graphene conductive layer. (**B**,**C**) Transmitted and SPR images of gold nanowires (1-μm AuNWs (red circled) and a 6-μm AuNW (blue circled)) [47].

Functionalized SPR biosensors with dielectric coatings and novel 2D materials have demonstrated enhanced capabilities in detecting surface charge distributions, surface electrochemical reactions, and molecular adsorption [46,47,48,49]. A prominent example is the integration of a dielectric layer to suppress unwanted Faradaic processes on the gold surface. As illustrated in Figure 4 of the referenced study, a gold film coated with a Cytop dielectric layer and a graphene conductive layer effectively reduces background electrochemical activity, enabling the precise imaging of localized surface reactions, such as those occurring on single gold nanowires [47].

**Figure 5:**
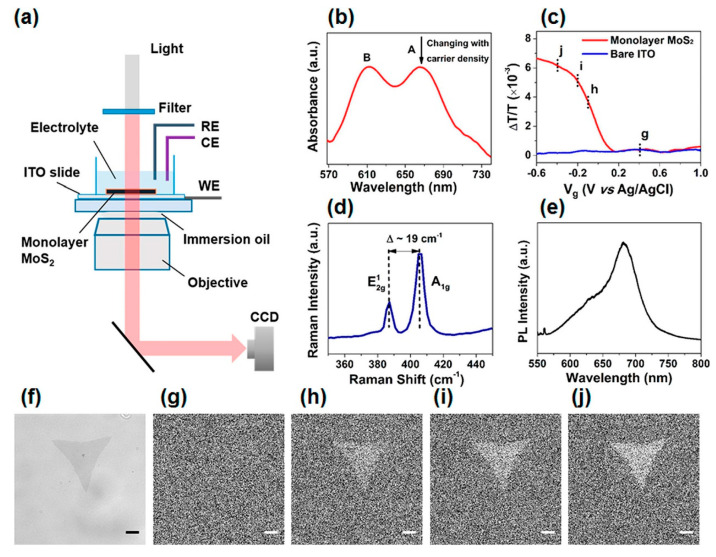
Optical imaging of local charge distributions using monolayer MoS_2_. (**a**) Schematic illustration of the setup. (**b**) Absorption spectrum of monolayer MoS_2_. (**c**) Transmission change as a function of gate voltage. (**d**) Raman spectrum of monolayer MoS_2_. (**e**) Photoluminescence spectrum. (**f**) Optical transmission image. (**g**–**j**) Differential images at various gate voltages. Scale bars: 5 μm [49].

Further advancements have been made by employing novel 2D materials, such as antimonene and molybdenum disulfide (MoS₂), which exhibit unique charge-sensitive optical properties. As depicted in Figure 5 of the referenced study, the experimental setup involves placing the monolayer MoS₂ on an indium tin oxide (ITO) slide and applying an electrochemical gate voltage to control the carrier density of the MoS₂ [49]. This configuration allows for the precise imaging of local charge distributions through optical transmission measurements. The system captures differential images at various gate voltages, revealing increased contrast with decreasing gate voltage, which is directly correlated with local charge density changes on the MoS₂ surface.

In addition to providing information on the molecular adsorption and orientation obtained from traditional methods, surface charge distribution offers additional insights. Traditional methods typically focus on the physical adsorption of molecules and how their spatial orientation affects binding efficiency [23,24,50]. However, surface charge distribution captures changes in the local electrostatic environment caused by molecular binding, such as charge transfer or shifts in surface potential. This provides valuable information about molecular interactions, particularly in cases involving charged molecules or electrochemically sensitive processes. While the molecular orientation explains how the alignment of molecules influences binding, the surface charge distribution reveals the electrostatic effects of these interactions [51,52,53]. By combining both aspects, biosensors can offer a more comprehensive and sensitive analysis of molecular behavior.

### 2.3. Optical Waveguide Biosensors

**Figure 6:**
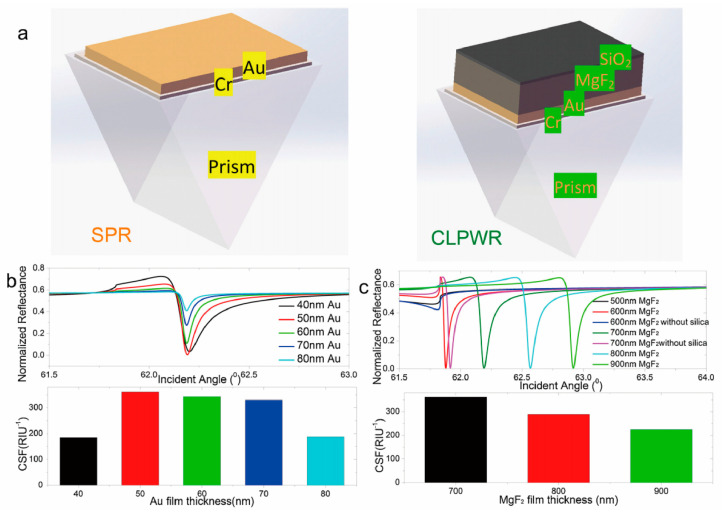
Optical waveguide biosensor. (**a**) Schematics of SPR and optical waveguide modules. (**b**) Calculated reflectance spectra and sensitivity factors with different Au film thicknesses. (**c**) Calculated reflectance spectra and sensitivity factors with different MgF_2_ film thicknesses [54].

Optical waveguide biosensors are typically constructed by depositing one or more dielectric layers—such as magnesium fluoride (MgF_2_) and silica—onto an SPR sensor chip to establish a dielectric sensing surface (Figure 6) [54]. When certain criteria are met, including the wavelength of the incident light, the angle of incidence, and the refractive index of the waveguide layer, optical waveguide modes are induced, generating surface waves on the waveguide layer for surface-sensitive measurements [55,56,57,58]. These biosensors are capable of producing a more pronounced resonance curve and a more intense field strength of surface waves compared to traditional SPR biosensors, thereby enhancing the resolution of detection.

### 2.4. Bloch Surface Wave Biosensors

**Figure 7:**
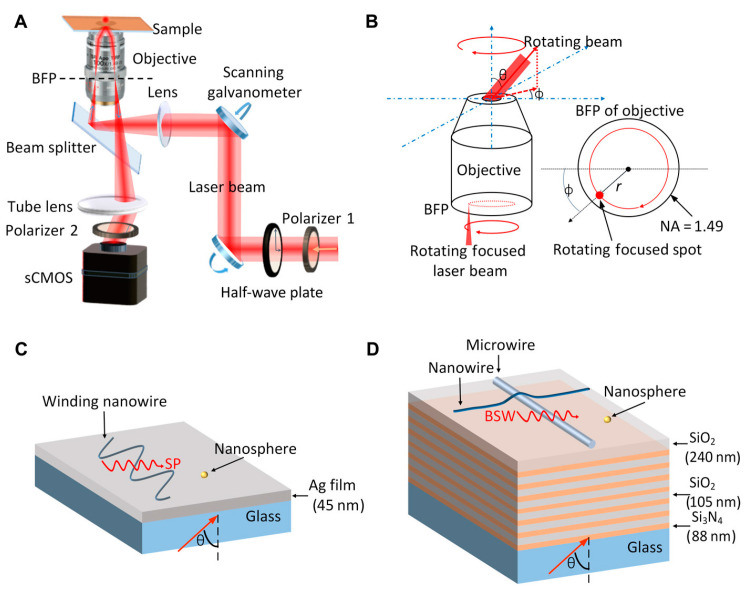
BSW biosensors. (**A**) Experimental setup. (**B**) System illumination configuration. (**C**,**D**) Substrates for SPR and BSW, respectively [61].

Bloch surface waves (BSWs) are electromagnetic waves that occur at the surface or interface of periodic dielectric structures, such as one-dimensional photonic crystals or dielectric multilayer films (Figure 7) [59,60,61,62,63,64]. These structures consist of alternating layers of materials with high and low refractive indices, creating a photonic bandgap that facilitates the excitation of BSWs at the upper dielectric interface. This setup eliminates the need for metallic layers, thereby reducing optical losses and boosting sensitivity [61]. The evanescent field produced by the BSW remains localized near the surface, which is advantageous for detecting slight refractive index changes due to biomolecular interactions. BSW biosensors leverage this surface wave phenomenon to perform surface-sensitive measurements on dielectric surfaces.

### 2.5. Photonic Crystal Resonator Biosensors

**Figure 8:**
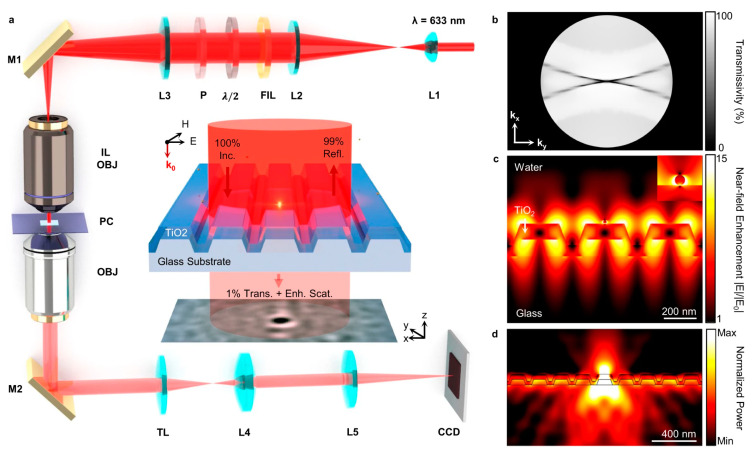
Photonic crystal resonator biosensors. (**a**) System sketch. (**b**) Simulated transmissivity at laser wavelength. (**c**) Near-field electric field profile of a gold nanoparticle. (**d**) Radiation power distribution of an electric dipole on the resonator surface [68].

Photonic crystal resonators are known for their ability to significantly enhance the surface light field [65,66,67]. When paired with an interferometric scattering detection scheme, these resonators enable the label-free imaging of single proteins using low-intensity incident light (Figure 8) [68]. By integrating a photonic crystal (PC) substrate into the microscopy system, a photonic crystal resonator biosensor can be constructed. This biosensor uses a TiO_2_-coated photonic crystal resonator to provide a dielectric surface. When exposed to a monochromatic laser, the PC utilizes photonic crystal-guided resonance to enhance light confinement and generate a surface wave suitable for surface-sensitive measurements [68]. This configuration amplifies the incident light more than 100 times, significantly increasing sensitivity and allowing for the precise detection of nano-objects such as proteins, viruses, and nanoparticles.

To provide a clear and concise understanding of the unique characteristics of different surface-based biosensors, we present a comparative analysis of several key sensor types. Table 1 outlines the working mechanisms, field enhancement, biocompatibility, and heat generation of these biosensors. By summarizing the advantages and disadvantages of each sensor, this table aims to assist researchers in selecting the most appropriate biosensing platform for their specific experimental needs.

## 3. Applications in Membrane Protein Binding Kinetics

### 3.1. Ensemble Measurements of Molecular Binding Kinetics in Buffer

SPR biosensors are extensively utilized for analyzing molecular binding kinetics, while surface-sensitive biosensors with dielectric surfaces have shown the capability to achieve greater measurement precision for quantifying molecular interactions, including those involving small molecules interacting with proteins [1,16,20,69].

**Figure 9:**
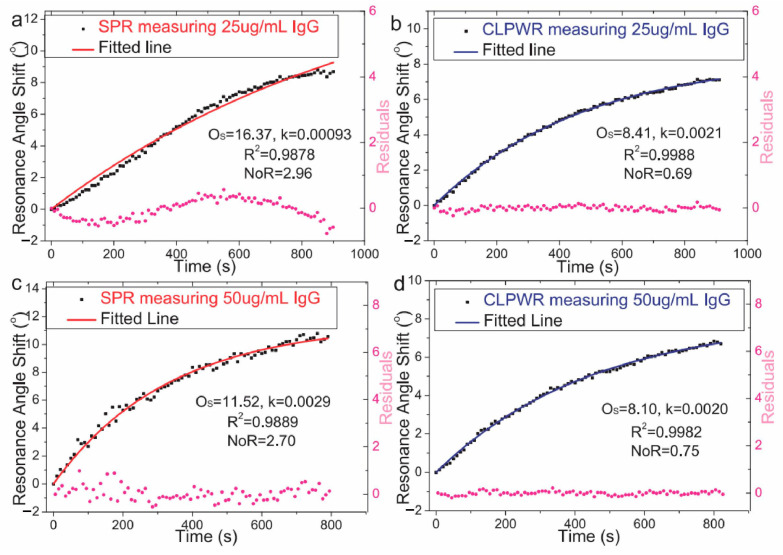
Optical waveguide biosensors detecting IgG binding. (**a**–**d**) Time course and fitting results for different IgG concentrations with the pink dots representing the residuals of the fit [54].

Figure 9 highlights the enhanced sensitivity of optical waveguide biosensors—a prime example of a surface-sensitive biosensor with a dielectric surface—in detecting the binding of IgG in human plasma to protein A receptors on the sensor surface. These optical waveguide biosensors offer more stable sensor outputs than SPR biosensors, resulting in smoother binding curves that facilitate more straightforward fitting to first-order binding kinetics models [54].

**Figure 10:**
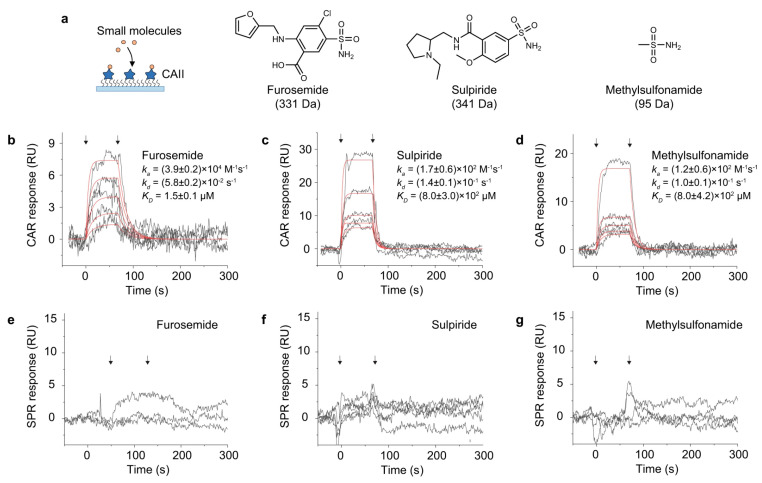
TIR-CAR biosensor measuring small molecule ligands binding to carbonic anhydrase II. (**a**) Immobilization of CAII on glass and gold surfaces. (**b**–**d**) CAR response curves for different ligands (black curves) and fitted results (red curves) interactions measured with SPR (the beginning and ending of binding phase is marked with arrow).(**e**–**g**) same interactions were measured wth SPR but no clear response was observed. The CAII surface coverages were 6.5% and 5.8% for the gold and the glass surfaces, respectively(the beginning and ending of binding phase is marked with arrow) [40].

In Figure 10, the TIR-CAR biosensor, another key type of surface-sensitive biosensor with a dielectric surface, demonstrates its ability to determine the binding kinetics of small molecules like furosemide, sulpiride, and methylsulfonamide to carbonic anhydrase II [40]. This is typically challenging for SPR biosensors. TIR-CAR biosensors enhance sensitivity for detecting subtle refractive index changes at the dielectric surface. The response curves for each ligand concentration were accurately fitted to first-order kinetics, enabling precise measurements of both the association and dissociation phases, which are vital for a comprehensive understanding of molecular interactions.

### 3.2. Digital Counting of Protein Binding Process

**Figure 11:**
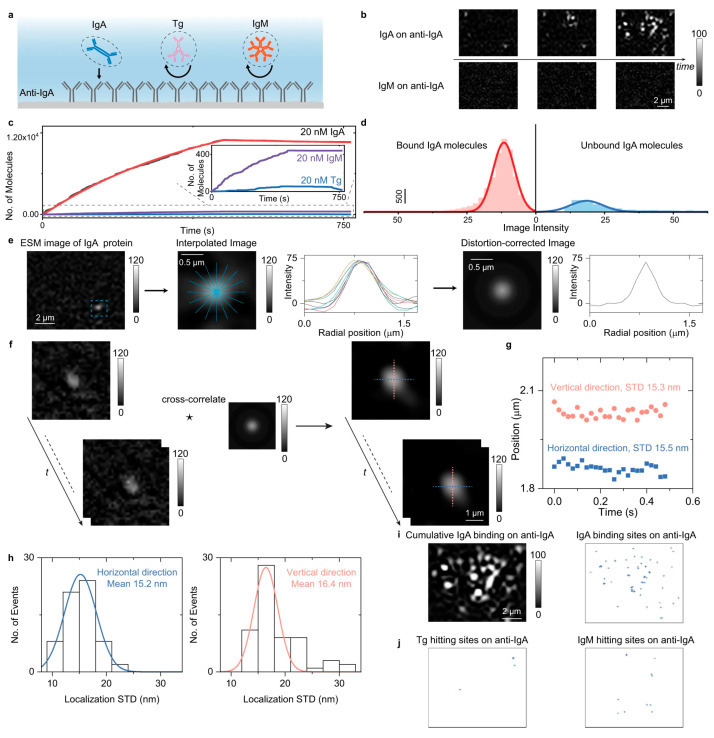
Digital counting of protein binding process with TIR-ESM. (**a**) Schematic of single-protein behaviors on modified surfaces. (**b**) ESM images of IgA binding and negative control. (**c**) Kinetics of IgA binding by digital counting. (**d**) Image intensity histogram. (**e**) Mean radial profiles for PSF construction. (**f**) Correcting ESM image distortions. (**g**) Tracking protein binding position. (**h**) Localization standard deviations. (**i**) Super-resolution image of IgA binding events. (**j**) Super-resolution image of Tg and IgM hitting events [40].

Combined with the scattering detection scheme, TIR-ESM permits single-protein imaging, making it possible to digitally count the protein binding events to evaluate the binding kinetics and the spatial distribution of binding events (Figure 11) [44,45]. This approach allows for the precise identification and counting of individual protein molecules, such as BSA, binding to the surface. By integrating multiple frames, the digital counting of protein binding and unbinding events provides a direct measurement of the kinetics. The data revealed that TIR-ESM could differentiate between specific and nonspecific binding interactions with a high spatial resolution. The ability to visualize and quantify binding kinetics at the single-molecule level, without requiring fluorescent labels or complicated systems, highlights TIR-ESM’s potential in offering high-precision, label-free kinetic analysis.

### 3.3. Tracking Object Movements Along the Z-Axis

**Figure 12:**
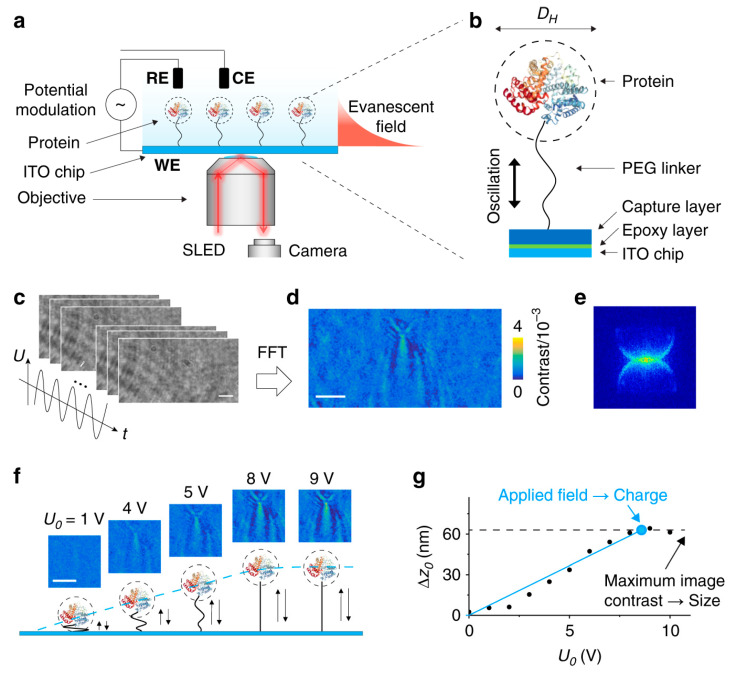
Imaging single proteins and mechanical oscillations. (**a**) Proteins tethered to an ITO surface and driven by an alternating electric field. (**b**) Time sequence images of oscillating molecules. (**c**) FFT image resolving a single BSA molecule. (**d**) Spatial Fourier Transform of the FFT image. (**e**) FFT image contrast vs. potential amplitude. (**f**) Oscillation amplitude of a BSA molecule vs. potential amplitude. (**g**) Oscillation amplitude (Δz_0_) of a BSA molecule vs. potential amplitude (U_0_) [70].

The evanescent field’s exponential decay characteristic significantly enhances the resolution along the *z*-axis perpendicular to the surface in surface-wave-based techniques, enabling the precise determination of a scatterer’s position along the *z*-axis based on its scattering intensity. Leveraging this attribute, nano-oscillators driven by electric field can be intricately engineered to measure the weight or charge of single-protein molecules utilizing an entropic elasticity model. Surface-sensitive biosensors with dielectric surfaces, such as TIR biosensors, offer minimal background Faraday signals in the electric field, facilitating higher contrast detection compared to SPR biosensors [70,71]. As illustrated in Figure 12, TIR biosensors can assess the oscillations of single proteins anchored to an ITO surface through polyethylene glycol (PEG) linkers, driven by an alternating electric field [70]. This setup enables the precise imaging of single-protein molecules oscillating in response to the field, thereby elucidating both the molecular size and charge. The scattering signal is analyzed using a fast Fourier Transform (FFT), which permits the accurate quantification of mechanical properties such as oscillation amplitude, size, and charge at the single-molecule level. This real-time detection technique offers unparalleled detail in the analysis of single-molecule protein dynamics, proving especially valuable for exploring molecular interactions and conformational changes.

### 3.4. In Situ Analysis of Membrane Protein Binding Kinetics and Multi-Parameter Extraction

**Figure 13:**
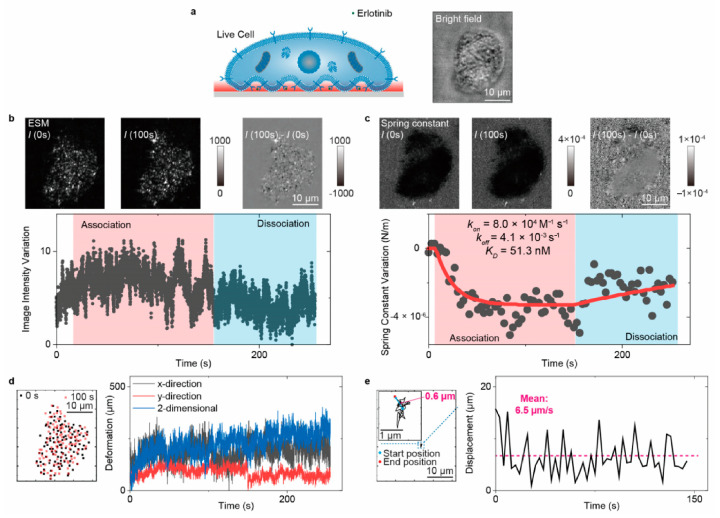
In situ analysis of membrane protein binding kinetics using TIR-ESM. (**a**) WGA binding to fixed A431 cells. (**b**) Raw and differential ESM images and intensity variation over time. (**c**) Spring constant maps and variation over time. (**d**) Cell adhesion site positions and deformation over time. (**e**) Cell center position movement traces [72].

**Figure 14:**
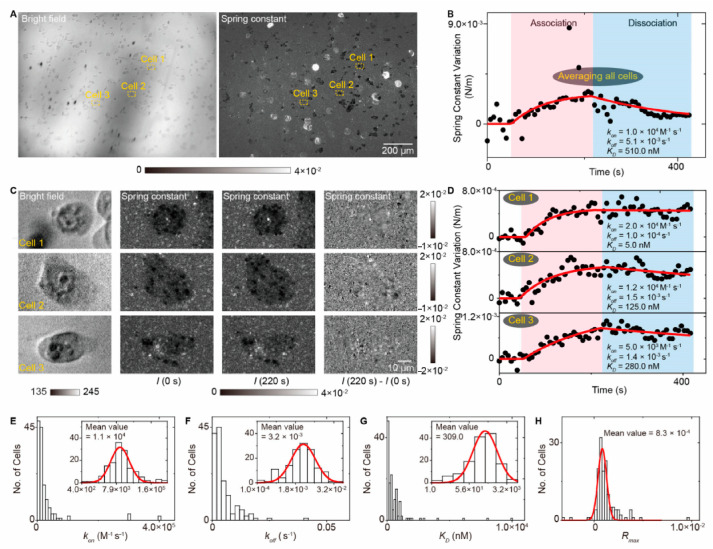
High-throughput single-cell analysis of membrane protein interactions. (**A**) Bright field image and spring constant map of live A431 cells. (**B**) Spring constant variation over time. (**C**) Zoomed views of cell regions at different times. (**D**) Spring constant variation from a marked cell. (**E**–**H**) Statistical distributions of binding parameters [73].

Given surface-wave-based imaging techniques’ ability to track object movements in an axial direction and introduce a statistical analysis approach to analyze the molecular binding conformations, the TIR biosensors can analyze the small molecule ligands interacting with the membrane protein in the living cells (Figure 13) [72]. Combined with large-view imaging systems, the TIR biosensors have shown the capability for high-throughput single-cell analysis (Figure 14) [73].

The study in Figure 13 uses TIR-ESM for the in situ analysis of the binding kinetics of erlotinib, a small molecule inhibitor, to the epidermal growth factor receptor (EGFR) on live A431 cells [72]. This setup not only tracks the binding events in real time but also allows for the simultaneous extraction of multiple parameters such as binding kinetics parameters and equilibrium constants. Moreover, the high *z*-axis resolution of the TIR biosensors plays a critical role in measuring the subtle movements of protein–ligand complexes, enabling the calculation of elasticity coefficients (spring constants) of the molecular bonds. Through this, TIR-ESM provides valuable insights into the mechanical properties of the interactions, such as the stiffness and tension of the membrane protein complexes.

In addition to providing detailed kinetic measurements, the integration of large field-of-view imaging systems significantly enhances the throughput of TIR-ESM biosensors for single-cell analysis. As shown in Figure 14, this setup enables the simultaneous monitoring of membrane protein interactions with small molecule ligands across multiple cells in a single view. The ability to analyze large numbers of cells in parallel while extracting multi-parameter data such as binding affinities, dissociation rates, and mechanical properties greatly improves the efficiency of membrane protein studies. This approach is particularly useful for examining heterogeneous cell populations, allowing for the quantification of cellular responses to ligands such as acetylcholine with high precision. By leveraging high-resolution imaging and real-time analysis, this system provides comprehensive insights into the dynamics of small molecule interactions with membrane proteins in living cells.

### 3.5. Multi-Protein Identification

**Figure 15:**
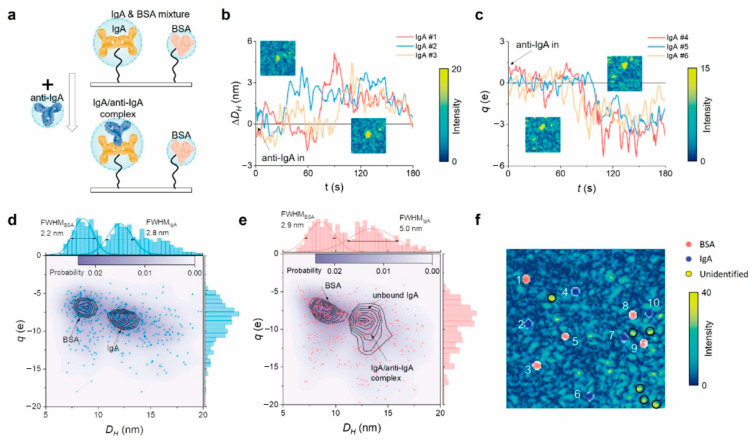
Identification of single proteins in a mixture. (**a**) IgA and BSA mixed and tethered to the surface. (**b**,**c**) Real-time monitoring of size and charge changes in IgA upon antibody binding. (**d**,**e**) Two-dimensional size and charge distribution before and after adding anti-IgA. (**f**) Example of identified IgA and BSA molecules [71].

Recent advancements in evanescent scattering techniques have enabled the simultaneous imaging of protein size and charge, which allows for the precise identification of individual proteins within heterogeneous mixtures [71,74]. As shown in Figure 15, this method captures distinct scattering profiles that reflect differences in size and charge, providing detailed insights into the unique binding characteristics of various proteins [71]. This approach not only identifies individual proteins but also quantifies their binding affinities and kinetic parameters, enabling a deeper understanding of protein–protein interactions at the single-molecule level. Such innovations are critical for elucidating complex molecular interactions relevant to health and disease.

**Figure 16:**
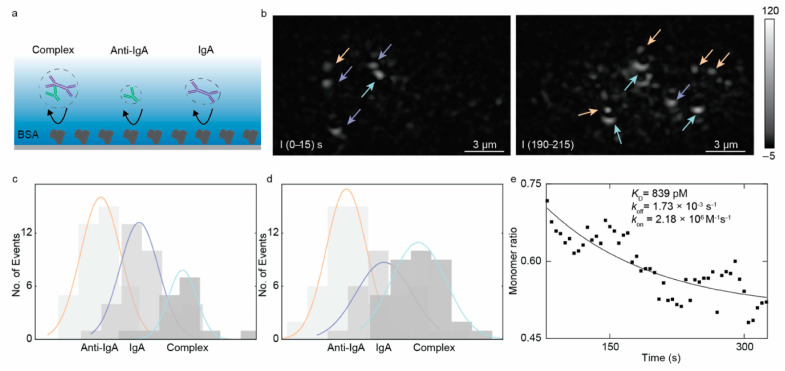
ESM detection of anti-IgA and IgA interactions. (**a**) Schematic of nonspecific bindings. (**b**) Protein (anti-IgA: orange arrow, IgA: purple arrow, and the complex: light green arrow) binding images at different times. (**c**,**d**) Histograms of protein counts over time and the fitted distribution curves with corresponding color in (**b**). (**e**) IgA monomer ratio as a function of time [74].

Evanescent Scattering Microscopy (ESM) provides a robust platform for multiplexed protein detection and parallel binding kinetics analysis. As illustrated in Figure 16, ESM enables the real-time tracking of protein interactions by distinguishing proteins of different molecular weights and analyzing their binding dynamics on the sensor surface [74]. This technique supports the simultaneous detection and kinetic analysis of multiple proteins, offering detailed insights into binding behaviors within complex biological samples. The use of differential processing to reduce background noise enhances the accuracy of detecting binding events, making ESM highly effective for applications such as biomarker screening and drug discovery.

### 3.6. Ultra-Sensitive Biomolecule Detection

**Figure 17:**
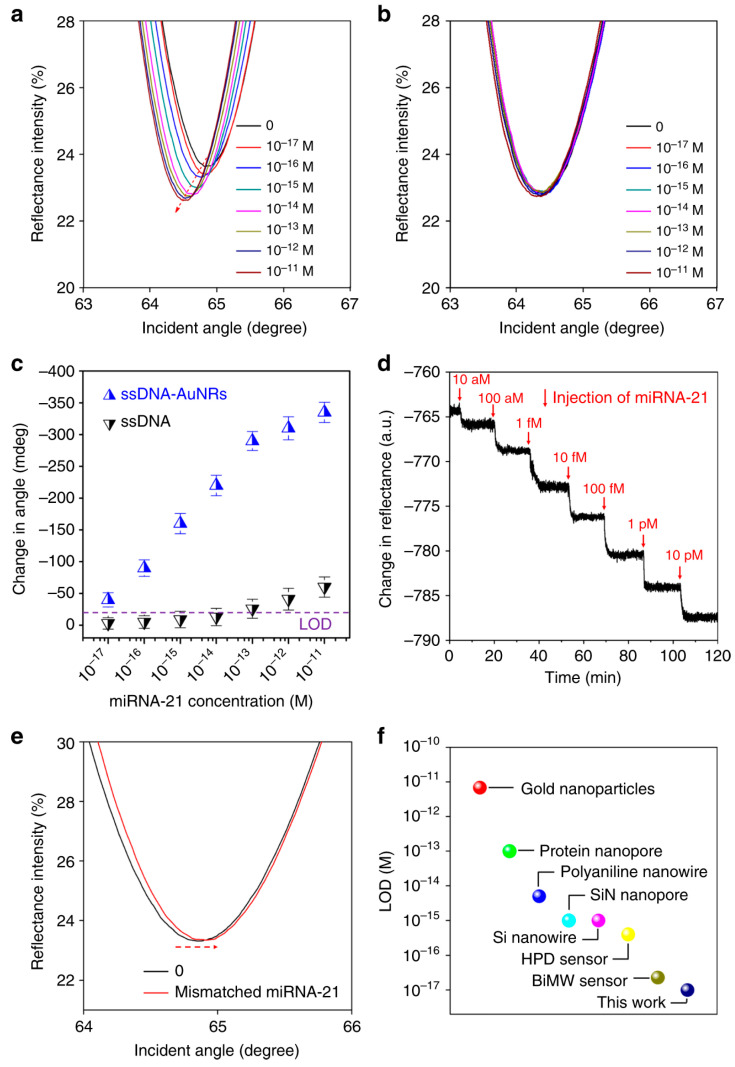
Ultra-sensitive detection of miRNA-21 using an antimonene SPR sensor. (**a**) SPR spectra with varying miRNA-21 concentrations. (**b**) Relationship between SPR angle and miRNA concentration. (**c**) Real-time SPR response of ssDNA-AuNR desorption. (**d**) SPR curve change with one mismatched nucleobase. (**e**) Comparison of detection limits. (**f**) Comparison of the LOD of the antimonene miRNA SPR sensor with that of state-of-the-art sensors [48].

Antimonene-based SPR sensors have demonstrated exceptional capabilities in detecting biomolecules at ultra-low concentrations, pushing the boundaries of sensitivity in biosensing. As depicted in Figure 17, the sensor achieves the highly sensitive detection of miRNA-21 with a detection limit reaching as low as 10 aM, which is significantly superior to conventional biosensors based on other 2D materials. The use of antimonene enhances the interaction with single-stranded DNA due to its strong adsorption energy and unique electronic properties, such as delocalized 5s/5p orbitals, which provide better sensitivity than traditional materials like graphene. This strong interaction facilitates the detection of biomolecular binding events, even at minute concentrations, making it particularly valuable for early disease diagnosis, including cancer.

In order to provide a comprehensive comparison of the different biosensing technologies discussed, the following Table 2 summarizes the test substance, binding kinetics, and refractive index resolution for various sensor types. These parameters are crucial for evaluating the effectiveness of biosensors in specific applications, such as high-sensitivity detection, molecular interaction analysis, and real-time monitoring. By compiling these performance metrics, the table offers insights into how each sensor performs under different experimental conditions, aiding researchers in selecting the most appropriate system for their needs.

## 4. Summary and Outlook

The burgeoning need for biomolecular interaction analysis has positioned SPR biosensors as a critical analytical tool, highly regarded for their label-free detection capabilities and exceptional surface sensitivity [7,8,75]. In pursuit of enhancing the performance and broadening the application spectrum of SPR biosensors, recent advancements have led to the development of surface-sensitive methodologies that incorporate dielectric surfaces. These dielectric surfaces offer several advantages: they can modulate surface waves to amplify field intensity, thereby elevating the sensitivity of measurements, and provide a biologically compatible interface that facilitates cellular attachment, enhancing the utility of SPR biosensors in cell-based assays. Moreover, surface-sensitive methods with dielectric surfaces can be easily integrated into existing SPR biosensor frameworks or microscopy systems, thus mitigating the challenges associated with technology adoption and integration. This compatibility allows for a seamless transition in upgrading conventional systems, making these advancements more accessible and reducing barriers to implementation in both research and clinical settings [76,77]. By leveraging dielectric surfaces, researchers can achieve more precise and sensitive biomolecular analyses, expanding the potential for these technologies in areas such as drug discovery, diagnostic development, and fundamental biological research [78,79].

Despite the advancements in surface-sensitive methods employing dielectric surfaces, these technologies are still in nascent stages of development and require further enhancement to fully meet the needs of biological and biochemical research communities. Firstly, innovative sensor designs are imperative to augment field intensity, thereby achieving an exceptionally high measurement sensitivity. While photonic crystal resonators have demonstrated the capacity to amplify the surface light field intensity by over 100 times, this enhancement is localized to specific “hot spots” rather than being distributed across a broader field. This limitation constrains their utility for wide-field imaging applications. The development of novel sensor chips that can sustain high-intensity surface waves across extensive areas remains a crucial objective. Secondly, dielectric surfaces offer significant benefits in facilitating the integration of various analytical approaches into surface-sensitive measurements as they help mitigate common issues like surface charging and fluorescence quenching observed with gold surfaces in traditional SPR biosensors. Looking ahead, the advancement of multimodal surface-sensitive imaging techniques represents a significant endeavor. Such methods would allow for multi-parametric analysis, providing a multifaceted evaluation of molecular interactions. This would enrich our understanding by enabling comprehensive and nuanced insights into the dynamics and mechanisms of these interactions, thereby propelling forward the fields of molecular biology, pharmacology, and diagnostics.

## Figures and Tables

**Table 1:** Comparison of mechanisms, field enhancement, biocompatibility, and heat generation of various surface-based biosensors.

Sensor Type	Mechanism	Field Enhancement	Biocompatibility	Heat Generation
Metallic SPR	Surface plasmon resonance on metallic surfaces	High	Requires modification	High
Optical waveguide biosensors	Confinement of light in waveguides	Very high	High	Moderate
Total internal reflection biosensors	Total internal reflection evanescent waves near surface	Moderate	High	Low
Bloch surface wave biosensors	Propagation of surface waves through alternating refractive index layers	Very high	High	Low
Photonic crystal resonator biosensors	Resonance effects in photonic crystal structures	Very high	High	Low

**Table 2:** Comparison of test substance, binding kinetics, and refractive index resolution for various biosensor technologies.

Methods	Test Substance	Binding Kinetics	Refractive Index Resolution	Reference
TIR-CAR	CAII with furosemide	k_a_ = (3.9 ± 0.2) × 10^4^ M^−1^s^−1^ k_d_ = (5.8 ± 0.2) × 10^−2^ s^−1^ K_D_ = 1.5 ± 0.1 μM	Not provided	[40]
	CAII with sulpiride	k_a_ = (1.7 ± 0.6) × 10^2^ M^−1^s^−1^ k_d_ = (1.4 ± 0.1) × 10^−1^ s^−1^ K_D_ = (8.0 ± 3.0) × 10^2^ μM		
	CAII with methylsulfonamide	k_a_ = (1.2 ± 0.6) × 10^2^ M^−1^s^−1^ k_d_ = (1.0 ± 0.1) × 10^−1^ s^−1^ K_D_ = (8.0 ± 4.2) × 10^2^ μM		
TIR-ESM	Live A431 cells with 25 μg/mL WGA	k_a_ = 1.5 × 10^4^ M^−1^s^−1^ k_d_ = 1.4 × 10^−3^ s^−1^ K_D_ = 0.093 μM	Not provided	[74]
	20 nM IgA with anti-IgA	k_a_ = 1.1 × 10^5^ M^−1^s^−1^ k_d_ = 9.6 × 10^−5^ s^−1^ K_D_ = 873 pM		
	Live A431 cells with 1 μM erlotinib	k_a_ = 1.0 × 10^4^ M^−1^s^−1^ k_d_ = 5.1 × 10^−3^ s^−1^ K_D_ = 0.51 μM		
SPR	Human plasma samples with 25 μg/mL IgG	K_D_ = 0.93 μM	9.2 × 10^−6^ RIU	[54]
	Human plasma samples with 50 μg/mL IgG	K_D_ = 2.9 μM		
CLPWR	Human plasma samples with 25 μg/mL IgG	K_D_ = 2.1 μM	8.3 × 10^−7^ RIU	[54]
	Human plasma samples with 50 μg/mL IgG	K_D_ = 2.0 μM		
